# Radiation Necrosis Following Proton Beam Therapy in the Pediatric Population: a Case Series

**DOI:** 10.7759/cureus.1785

**Published:** 2017-10-19

**Authors:** Justin Davanzo, Robert J Greiner, Mustafa Barbour, Elias Rizk

**Affiliations:** 1 Department of Neurosurgery, Penn State Milton S. Hershey Medical Center; 2 Pediatric Oncology, Penn State Milton S. Hershey Medical Center

**Keywords:** radiation necrosis, proton therapy, pediatric neurosurgery, pediatric neuro-oncology

## Abstract

Radiation necrosis after proton beam radiotherapy in the pediatric population is a finding that should be evaluated. We present two cases of radiation necrosis in pediatric patients who underwent proton beam radiation therapy following gross total resection of tumors. As seen in both our cases, patients often present with radiographic changes found on surveillance imaging. While the progression of disease should certainly be considered in any patient with radiographic changes, understanding the radiographic findings and the clinical course of radiation necrosis is paramount in order to prevent unnecessary surgical intervention.

## Introduction

Radiation necrosis is a well-described phenomenon that has been illustrated with a multitude of radiation therapies at a wide range of doses [1]. While biopsy of these lesions is possible, an accurate diagnosis is often made through a combination of clinical and radiographic factors. Most available data surrounding radiation necrosis is from the adult population; however, radiation necrosis is an occurrence that has been similarly noted in children, including those who received proton beam radiotherapy [2]. We present a case series of two patients with radiation necrosis after proton beam radiotherapy.

## Case presentation

**Figure 1:**
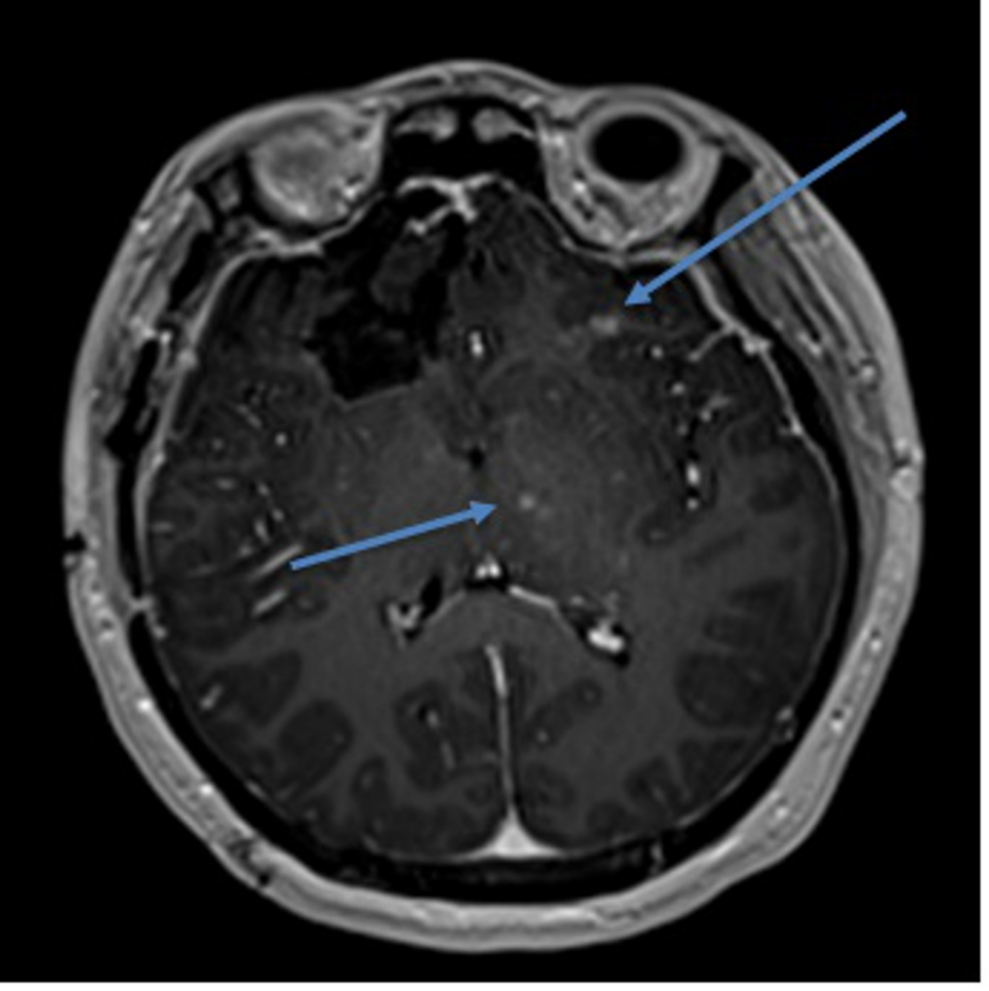
T1-weighted brain magnetic resonance imaging with contrast Arrows indicate areas of enhancement distant from the surgical field.

Our first patient is a 14-year-old female who presented to the neurosurgery service with an enhancing frontal lesion near the end of December 2013. She underwent gross total resection; pathology was found to be anaplastic ependymoma. The patient subsequently underwent focal proton beam radiation therapy, 50.4 Gray (Gy) in 28 fractions; additionally, she was closely followed by Neuro-Oncology and Neurosurgery. The magnetic resonance imaging (MRI) at her one-year post-operative screening showed multiple areas of enhancement (Figure 1). Her team, including Neurosurgery and Pediatric Hematology-Oncology, determined that while these lesions may be located within the radiation field, they were distant enough from the original resection cavity that biopsy was warranted. Stereotactic biopsy was performed which exhibited reactive changes, consistent with radiation necrosis and not of recurrence of neoplastic lesion. The patient was followed with serial imaging. These lesions continued to improve radiographically with each follow-up image. No changes were made to the patient’s treatment regimen. Ultimately, complete radiographic resolution was achieved, and the patient remained asymptomatic.

**Figure 2:**
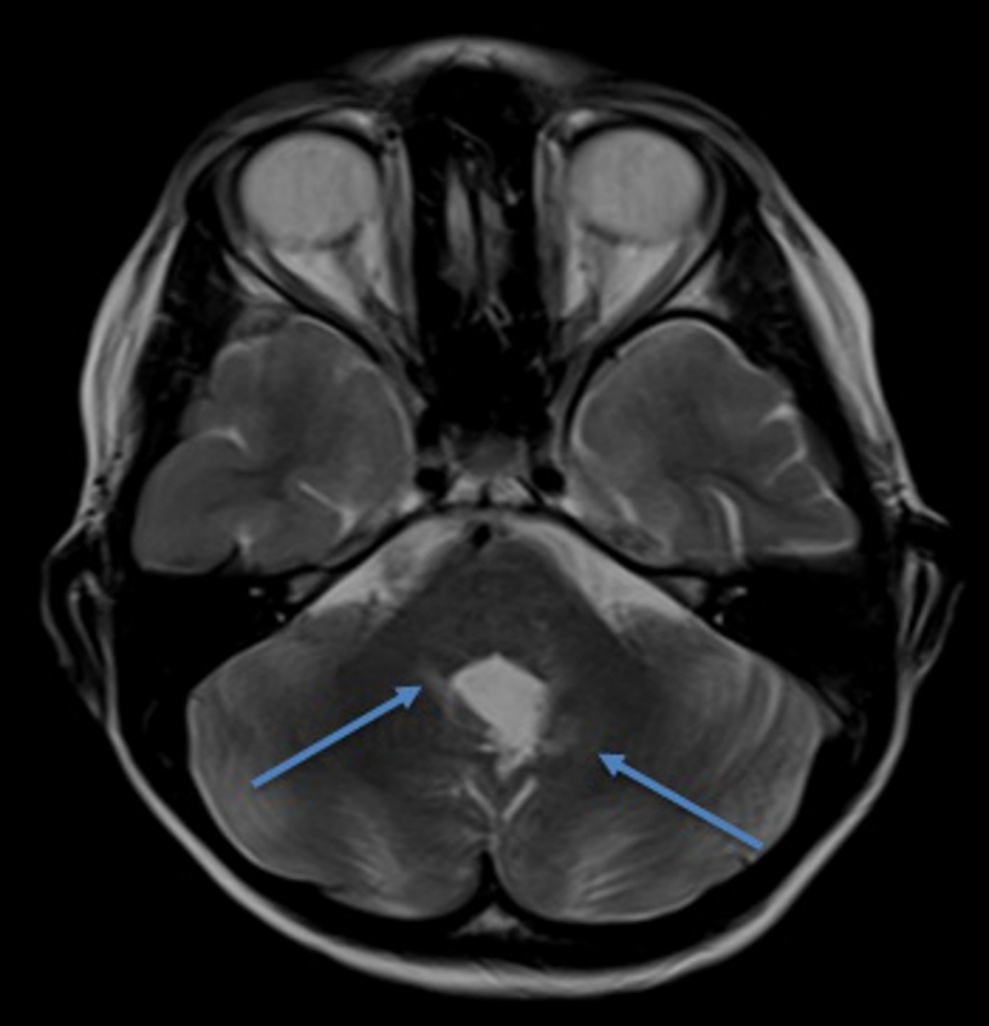
T2-weighted brain magnetic resonance imaging Arrows indicate areas of hyperintensity distant from the surgical field.

Our second patient is a nine-year-old male who was diagnosed with a posterior fossa lesion in September of 2014. He underwent gross total resection; pathology revealed medulloblastoma. He subsequently underwent craniospinal proton beam radiation therapy (23.4 Gy to the craniospinal axis and a conformal tumor boost resulting in a total posterior fossa dose of 54 Gy). On his six-month post-operative screening MRI, he was noted to have some areas of T2 and fluid-attenuated inversion recovery (FLAIR) hyperintensity near the region of radiation (Figure 2). These changes were not consistent with tumor recurrence and much more likely to be radiation changes. The patient was followed with serial imaging; no changes were made to his treatment regimen based on these findings. Once again, with each follow-up image, these areas were noted to be resolving radiographically. The patient experienced no symptoms from these lesions and ultimately achieved complete radiographic resolution.

## Discussion

Our small series presents two patients who both underwent proton beam radiation therapy and who similarly developed subsequent radiographic changes. Our first patient had changes that were distant from the resection cavity; thus, a biopsy was taken and the diagnosis of radiation necrosis was confirmed. As Kralik, et al. stated in their findings, enhancing lesions some distance away from the surgical field is a common imaging finding in radiation necrosis after proton beam therapy [3]. Both patients continued to undergo serial imaging after these findings were noted. We found that both patients’ radiographic findings diminished over time, with no alteration in their neoplastic-directed therapy.

Radiation necrosis is a phenomenon that was first described by Fischer and Holfelder in 1930 [4]. They reported changes after radiotherapy was delivered for a basal cell carcinoma of the scalp. Since their report, multiple case series and studies have shown this finding in a wide variety of neoplastic lesions, utilizing distinct radiation treatment protocols. Several of these available reports consist of patients who received proton beam radiation therapy [1, 3, 5].

Clinically, patients may be symptomatic or asymptomatic in conjunction with these imaging findings. Plimpton, et al. reported that five out of 101 patients developed imaging changes consistent with radiation necrosis; however, only three of these five patients were found to have symptoms as a result of these radiographic changes [6]. Also, Indelicato, et al. reported brainstem toxicity on a large series of pediatric patients who had undergone proton beam radiotherapy. In this study, brainstem toxicity was defined by new clinical symptoms and corresponding radiographic findings. They showed that 11 of the 313 patients experienced brainstem toxicity after proton beam radiotherapy, all of whom had neoplasms located within the posterior fossa [7]. As with most cerebral pathology, the location of the lesion plays a large role in determining whether the patient becomes symptomatic.

Although a somewhat rare occurrence, radiation necrosis following radiation therapy, including proton beam radiation therapy, is a diagnosis that should be considered in the pediatric population. Unfortunately, this is not a simple diagnosis based on radiographic or clinical information. Radiographically, the changes seen in radiation necrosis are oftentimes subtle. Fouladi, et al. created a grading system for radiation-induced changes noted on MRIs in pediatric populations: Grade 1 was were those that displayed changes on T2/FLAIR imaging, Grade 2 included Grade 1 changes but also with contrast enhancement on T1 imaging, Grade 3 lesions were those that showed evidence of hemorrhage, and Grade 4 was found to have encephalomalacia [8]. Clearly, Grade 1 and Grade 2 lesions can be subtle, with potentially misleading findings on post-operative MRI imaging. Other studies have noted that the most common findings in radiation necrosis in pediatric patients were multiple small areas of enhancement distant from the site of surgical resection [3, 5].

Many researchers have attempted to determine which, if any, clinical factors increase the patient’s likelihood of developing radiation necrosis. This determination has proven challenging due, in part, to the low number of pediatric patients that develop this complication. Kralik, et al. were able to determine that, in their 60 patient series, those who received more than three chemotherapeutic agents, and those who were found to have atypical teratoid rhabdoid tumor (ATRT) as their pathologic diagnosis had significantly higher rates of radiation necrosis. They did not, however, find any statistically significant relationship with regards to age, sex, gross total versus subtotal resection, or even radiation dose [3]. Gunther, et al. also showed that patients who underwent proton beam radiotherapy had a higher incidence of radiographic changes which resolved without changes in treatment. They also noted that these changes tended to occur more frequently in those patients under three years of age [2]. It should be noted that several other studies also indicate that increased radiation doses are associated with increased rates of radiation necrosis; however, all of these have been in the adult population [9-10].

The discovery of the disease in the pediatric population is an important one and should not be dismissed. Currently, standard practice is to perform surveillance cranial imaging of neoplastic lesions. As a result, many asymptomatic imaging changes will be noted. While these certainly could represent progression of disease, one must consider that these changes could also represent radiation necrosis. Understanding this difference is crucial information because radiation necrosis usually resolves without surgical intervention.

## Conclusions

Understanding the radiographic findings and the clinical course of radiation necrosis is paramount. By understanding the radiographic findings and clinical course of radiation necrosis, neurosurgeons can avoid unnecessary surgical intervention. In addition, it is important to understand the relationship of proton beam radiotherapy and radiation necrosis. It has been noted in both the available literature and in our patient series that this tends to occur outside of the surgical resection cavity, occurring early after treatment. Once again, this is important, because lesions which appear distant from the initial site of the lesion are certainly concerning for recurrence. However, if this finding occurs shortly after proton beam radiotherapy, it may be reasonable to observe these closely.
